# Feasibility of alternating induction and maintenance chemotherapy in pancreatic cancer

**DOI:** 10.1038/srep41549

**Published:** 2017-01-31

**Authors:** Alexander Hann, Wolfram Bohle, Jan Egger, Wolfram Zoller

**Affiliations:** 1 Department of General Internal Medicine and Gastroenterology, Katharinenhospital, Stuttgart, Germany; 2 Department of Internal Medicine I, Ulm University, Ulm, Germany; 3 Institute for Computer Graphics and Vision, Graz University of Technology, Austria

## Abstract

Chemotherapy regimens for pancreatic ductal adenocarcinoma (PDAC) have changed since the introduction of FOLFIRINOX. Due to toxicity, dosage and number of applied cycles are limited. In analogy to chemotherapy strategies in colon cancer we used a scheme of induction, maintenance and re-induction therapy in PDAC to alleviate such toxicities and increase the number of applied cycles. Here we report first experiences with this approach. Data of all patients who received FOLFIRINOX for metastatic or locally advanced PDAC in our center using induction chemotherapy followed by maintenance therapy from 2011 until November 2016 was collected and analyzed retrospectively. Progression free survival was assessed starting induction therapy until progressive disease (PD) during maintenance or treatment pause (PFS1) and until progression during re-induction therapy (PFS2). 13 patients received induction therapy which was followed by maintenance therapy. Re-induction due to PD during therapy was applied in 11 patients. The median PFS1 was 10.6 months (95% CI; 6.7–14.4), PFS2 was 14.1 months (95% CI; 8.2–19.9) and overall survival was 18.3 months (95% CI; 14.8–21.8). The use of FOLFIRINOX as induction, followed by maintenance and re-induction therapy in case of PD is feasible in the treatment of PDAC and might lead to a prolonged PFS with less toxicity.

The introduction of FOLFIRINOX in the treatment of metastatic pancreatic ductal adenocarcinoma (PDAC) was associated with a marked increase in survival^1^. The use of this new drug combination rapidly replaced gemcitabine as a first line chemotherapy in the palliative treatment of patients with good ECOG status. Approximately half of the first line chemotherapy used in our center consists of FOLFIRINOX^2^. But usage of this new chemotherapy regimen is also associated with increased toxicity. Especially neutropenia and oxaliplatin-related peripheral neuropathy are limiting factors for the continuation of this therapy. Due to the increased survival of patients with PDAC, strategies for prolonging time on chemotherapy to control tumor growth are needed. In colorectal cancer these chemotherapeutic substances are well known and different strategies were developed to cope with such toxicities. In the OPTIMOX 1 trial a chemotherapy regimen including 5FU/LV and oxaliplatin (FOLFOX) was evaluated in two different administration strategies^3^. One included FOLFOX, which was administered until progression or occurrence of unacceptable toxicity (Group A). The comparing regimen included also the usage of intensive treatment with both active drugs (FOLFOX) followed by a planned de-escalation after six cycles and a maintenance period with only 5FU/LV (Group B). In case of progressive disease reintroduction of the second active drug was started to regain disease control. The results presented a comparable outcome in regard to progression free survival (Group A: 9 months vs. Group B: 8.7 months) and overall survival (Group A: 19.3 months vs. Group B: 21.2 months). Another recently published study in colon cancer patients used a combination of capecitabine and bevacizumab as maintenance therapy. Induction therapy additionally included oxaliplatin (CAPOX-B). In this study the control arm included patients who were only observed after induction therapy and received no further treatment. In case of progression, patients received re-induction therapy (CAPOX-B) until second progression (PFS2). The intent-to-treat analysis presented a significant improvement in PFS2 in patients receiving maintenance therapy in comparison to observation (11.7 vs. 8.5 months, p < 0.0001)^4^. Further studies supported the use of maintenance therapy after an induction period containing multiple active chemotherapeutic drugs^5^,^6^. The recommendation of such maintenance and re-induction strategies are part of national treatment guidelines for colon cancer^7^. We believe that such strategies may be applicable to pancreatic cancer and may improve the tolerability of chemotherapy without compromising anti-tumor effect. Our clinical management includes an induction chemotherapy with FOLFIRINOX that can be deescalated during clinical course to FOLFOX in case of poor tolerability. In case of non-progression a maintenance therapy with 5FU/LV is usually started after the sixth cycle. Maintenance therapy is administered until progression. Some patients receive treatment pause during maintenance therapy predominantly based on patient’s wishes. In case of PD during maintenance or treatment pause patients are re-induced with FOLFIRINOX or FOLFOX.

In this retrospective study we describe our experiences with the use of this novel scheme of induction, maintenance, treatment pause and re-induction of chemotherapeutic drugs included in FOLFIRINOX in patients with PDAC.

## Results

### Patient characteristics

Between 2011 and February 2016 65 Patients were diagnosed with PDAC and treated with FOLFIRINOX at our institution. 13 patients met the inclusion criteria consisting of induction chemotherapy followed by 5FU/LV maintenance therapy. 43 patients were included as a control group. These patients started with FOLFIRINOX, but 27 did not receive other second line substances. The other 16 patients received a gemcitabine based second line chemotherapy after FOLFIRINOX. Nine patients were excluded because FOLFIRINOX was not applied as a first line palliative treatment. Most of these patients received FOLFIRINOX as a second, or third line palliative treatment. Demographic baseline characteristics were balanced between the two groups. They presented a higher proportion of male patients. Most of the treated patients had synchronous metastasis at diagnosis (Table 1). The treatment course of each patient in the 5FU/LV maintenance group regarding the applied number of chemotherapy cycles is depicted in Table 2. Staging frequency was approximately every 2 to 3 months.

### Induction chemotherapy

Induction chemotherapy starting with FOLFIRINOX was performed in all 13 patients. It had to be de-escalated to FOLFOX in 6 patients to increase the tolerability. De-escalation was done due to leukopenia in four and due to nausea in two patients. The de-escalated induction therapy was applied after a median of 3 cycles FOLFIRINOX (range 1–5) and consisted of additional median 4 cycles of FOLFOX. The median number of delivered chemotherapy cycles during induction using FOLFIRINOX and FOLFOX was 6 (range 5–13) (Table 3). About one fifth of all applied cycles during induction were administered with a reduced dosage. Oxaliplatin with a dosage of 75% due to neuropathy represented almost all of these changes. Induction therapy was applied during a median time of 19.9 weeks (range 12.3–27.3).

### Maintenance chemotherapy and treatment pause

**Figure 1:**
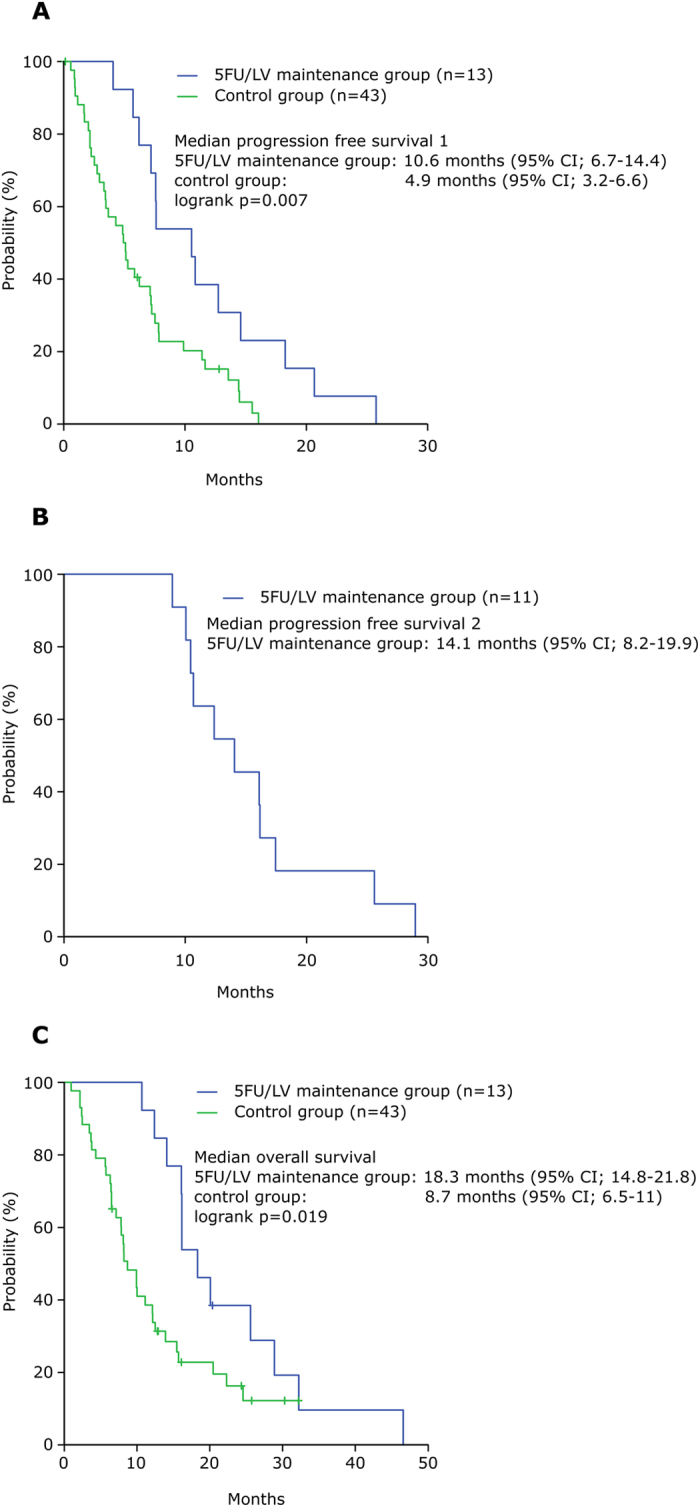
PFS1, PFS2 and OS.

After the induction chemotherapy all patients received maintenance therapy with 5FU/LV (Table 4). De-escalation of induction chemotherapy to maintenance was done in six patients due to good response or to prevent toxic effects, three patients were de-escalated due to leukopenia, two due to neuropathy, one due to emesis and one due to diarrhea. A median of 6 (range 2–21) cycles was administered. Dose reduction was necessary in fewer cases necessary compared to induction therapy. Four patients underwent treatment pause after receiving a median of 5 (range 2–21) cycles (Table 5). The introduction of a treatment pause in all cases was due to patient wish. Interestingly only patients that received induction therapy exclusively with FOLFIRINOX had treatment pause during maintenance. The PFS1 representing the time until progression during maintenance or treatment pause was 10.6 months (95% CI; 6.7–14.4 months) (Fig. 1). Comparing the PFS1 of the 5FU/LV maintenance group with the patients treated in the control group revealed a significant difference of 5.7 months. One possible explanation could be that patients with progressive disease during FOLFIRINOX treatment did not receive maintenance therapy with 5FU/LV.

### Re-induction chemotherapy

Due to progressive disease during maintenance or treatment pause 11 patients (85%) received re-induction chemotherapy (Table 6). Five patients received FOLFOX and six patients FOLFIRINOX as re-induction. The number of applied cycles was less in the re-induction therapy compared to the induction period. Additionally, more delay of cycles occurred during re-induction (34% vs. 16%). The PFS2, representing the time until progression during re-induction, was 14.1 months (95% CI; 8.2–19.9 months). The overall survival (OS) starting at the beginning of palliative chemotherapy was 18.3 months (95% CI; 14.8–21.8 months) (Fig. 1). The OS was significantly higher in the 5FU/LV maintenance group with 18.3 months (95% CI; 14.8–21.8) compared to the control group with 8.7 months (95% CI; 6.5–11).

### Adverse effects

Side effects that altered the management of treatment were documented in 48 cases of the total 243 applied chemotherapy cycles (Table 7). Most of the events were due to leukopenia of less than 3.0 × 10^3^/μL. Neuropathy was the second most recorded event and was a main reason for reducing the dosage of oxaliplatin. The extend of the neuropathy was estimated using the documentation of every visit. It was often paresthesia (CTCAE v4.03 grade I) or limited instrumental activities of daily living (ADL) (CTCAE grade II), although only grade II toxicity altered the management of treatment. Neuropathy that limited self-care ADL (CTCAE grade III) was never described by any patient. This reflects that 81% of re-induction chemotherapy cycles were applied at a full dose. The oxaliplatin dose was never reduced below 75%. Three hospitalizations that led to a delay in the application of the next chemotherapy cycle occurred. One was due to cholangitis. Two were due to pleural effusion.

## Discussion

The survival of patients with PDAC is gradually increasing most likely due to the combination of new chemotherapeutic agents^1^,^8^. As we have previously shown, the usage of FOLFIRINOX plays an important role in our center since its introduction in 2011. Since then we observed a marked increase in survival of our patients with PDAC receiving palliative chemotherapy^2^. As a result of the prolonged survival new strategies that address the toxicities associated with FOLFIRINOX are needed. Such strategies are well established in the therapy of colon cancer and are based on induction, maintenance and re-induction therapy^3^. In this retrospective study we present our first experiences with such strategies in PDAC treatment, using only substances included in FOLFIRINOX. We are able to demonstrate the feasibility of this regimen in the palliative treatment of PDAC patients that resulted in a remarkable progression free survival of 10.6 months until first progression. The patients treated with FOLFIRINOX in the PRODIGE/ACCORD 11 trial published by Conroy *et al*. showed a median PFS of 6.4 months (95% CI, 5.5–7.2)^1^. This difference was achieved although the patients treated in our study received fewer cycles of induction therapy (median number of cycles 6 (range 5–13) vs. 10 (range 1–47)). Due to the only moderately toxic maintenance therapy, a prolonged period of time on chemotherapy with a mean of 6 cycles (range 2–21) could be achieved until first progression. Adding up the number of induction and maintenance therapy cycles results in a median number of 15 cycles (range 8–25). Furthermore, using treatment pause after obtaining a stabilization of the disease during maintenance seems not to have negatively influenced the prognosis of the patients regarding progression free survival. Four patients received a treatment pause during maintenance therapy with successful re-induction in all cases. The PFS1 of patients receiving treatment pause was 10.6 months compared to 7.6 months of patients without pause (p = 0.6). In total 85% of the patients were able to receive re-induction chemotherapy. Using re-induction chemotherapy allowed for an increase in progression free survival to PFS2 of 14.1 months. Additionally, the OS was remarkably long for PDAC patients receiving palliative chemotherapy with 18.3 months. Comparing these results to the control group revealed a significant difference regarding PFS1 and OS with a benefit favoring the new scheme of median 5.7 months and 9.6 months respectively. An explanation for the improved clinical outcome of the 5FU/LV maintenance group might be that patients who had progressive disease during induction, or did not tolerate the induction chemotherapy, could not receive maintenance therapy.

Another advantage of the presented chemotherapy application strategy is that the therapy consisted only of substances included in FOLFIRINOX. Thus other second line substances have not been used in these patients. Eight out of 13 patients (62%) received a second line therapy. Five patients received a gemcitabine based chemotherapy, two 5FU/LV and one radio chemotherapy. Thus the rate of second line therapy is higher than the rate in the MPACT or PRODIGE/ACCORD 11 trials of 42 to 47%^1^,^8^.

Chemotherapy was well tolerated with leukopenia grade II (white blood cell count 2.0 to 3.0 × 10^3^/μL) being the main adverse event that lead mostly to treatment delay. The rate of side effects seen in this study is comparable to data reported by other institutions^9^,^10^,^11^. A reduction mainly of hematologic side effects might have been achieved by exclusion of the 5FU bolus^12^.

A thorough search of the literature revealed just a recently published retrospective single center analysis of Reure and colleagues^13^. The authors describe a similar protocol consisting of induction chemotherapy using FOLFIRINOX. Capecitabine as a 5FU prodrug was chosen as maintenance therapy after induction and was applied until progression. The OS of 17 months was comparable to the survival described in this manuscript of 18.3 months. Capecitabine is advantageous for the patients due to its oral availability in comparison to infusional 5FU. In our clinical practice patients using capecitabine often complain about side effects which lead to dose reductions. One main side effect is the hand-foot syndrome, which was seen with CTCAE grade 3 or 4 in 16.6%. No hand-foot syndrome was described in our study population. In the study of Reure, the dose of capecitabine maintenance therapy was reduced due to side effects in 35%, compared to only 9% with infusional 5FU/LV described in our collective. Additionally, this might have resulted in the different PFS1, which was 5 months using FOLFIRINOX and capecitabine compared to 10.6 months in our study. The rate of re-induction chemotherapy using an oxaliplatin-containing regimen (FOLFIRINOX or FOLFOX) was also lower in the study of Reure and colleagues with approximately 50% compared to 85% in our patients. Nonetheless we believe that both concepts are effective and feasible, but prospective studies that asses the benefit of such strategies in PDAC patients are needed.

Some weak points of this study are due to its retrospective nature. Usually this de-escalation of therapy is discussed with every patient after the sixth cycle. Due to inclusion of the patient’s wishes in the decision making process, some patients received more than 6 cycles of induction therapy. Only 4 out of 13 patients received more than 8 cycles induction therapy. Additionally, we used FOLFOX as a deescalated induction chemotherapy to improve FOLFIRINOX related side effects other that peripheral neuropathy. This regimen was shown to be effective in the second line therapy, but represents no first line therapy. In our experience the combination of 5FU/LV with oxaliplatin is very effective, like it was shown in previous studies for use as a second line therapy^14^,^15^.

The adverse events were not recorded prospectively, so there may be an underestimation of the amount of events that occurred. Examples are events that did not alter chemotherapy dosage or the time point of the next cycle. Additionally, compared to the total number of patients that received FOLFIRINOX during the study period, only 20% were treated with induction chemotherapy followed by maintenance therapy. Thus, this study population represents a highly selected group. This selection might have enriched patients with factors that positively influence survival or response to therapy. One example is the BRCAness gene signature which is currently under evaluation as a potential target for Olaparib (ClinicalTrials.gov Identifier: NCT02677038). This gene signature leads to a higher susceptibility of the tumor for platin-based chemotherapy^16^. Additionally quality of life (QoL) was not assessed due to the retrospective nature, but would be an important parameter in a prospective study. A better QoL without compromising efficacy would be very advantageous.

Nonetheless we hypothesize that the use of induction treatment with FOLFIRINOX, followed by maintenance therapy with the option of treatment pause and re-induction in case of progressive disease is feasible in the palliative treatment of PDAC patients.

To further investigate the benefits of this chemotherapy regimen prospective studies are needed.

## Methods

We identified patients with a histologically confirmed PDAC that were treated at the department of Gastroenterology, Katharinenhospital, Stuttgart, Germany, with FOLFIRINOX from 2011 to February 2016. Inclusion criteria were induction therapy with FOLFIRINOX with optional subsequent de-escalated induction therapy consisting of 5FU/LV combined with oxaliplatin (FOLFOX), followed by maintenance therapy consisting of 5FU/LV. Response to therapy and toxicity of the treatment were assessed until November 2016. Patients who received FOLFIRINOX as a first line palliative treatment but did not receive 5FU/LV maintenance therapy were selected as a control group. A minimum of 8 weeks was defined as treatment pause. Re-induction was defined as chemotherapy after maintenance or treatment pause of at least 8 weeks. Re-induction chemotherapies included FOLFIRINOX and FOLFOX, depending on previous therapy and toxicity. A regular full dose of FOLFIRINOX was identical to the PRODIGE/ACCORD-11 trial^1^ and consisted of oxaliplatin 85 mg/m^2^ over 2 hours, irinotecan 180 mg/m^2^ over 90 minutes, leucovorin 400 mg/m^2^ over 2 hours, 5-FU 400 mg/m^2^ bolus and 5FU 2400 mg/m^2^ over 46 hours continuous infusion with adequate supportive care. Each cycle was scheduled to be repeated after 14 days. The FOLFOX regimen consisted of the same substances included in FOLFIRINOX except irinotecan^14^. 5FU/LV consisted of leucovorin 400 mg/m^2^ over 2 hours, 5-FU 400 mg/m^2^ bolus and 5FU 2400 mg/m^2^ over 46 hours continuous infusion^17^.

Progression free survival was assessed starting at the beginning of the induction therapy until progression during maintenance therapy or treatment pause (PFS1) and until progression during re-induction (PFS2) (Supplementary Figure 1). PFS was assessed in the control group starting at the beginning of FOLFIRINOX until progression. Overall survival was assessed starting at the beginning of chemotherapy with FOLFIRINOX. Only those side effects of chemotherapy were assessed which altered the management of treatment. These events included discontinuation of the chemotherapy, delay of the next cycle of more than six days and dose reduction. Progression free survival was estimated with the Kaplan Meier method using IBM SPSS Statistics 23. The local ethical committee provided a waiver of the requirement for informed consent for this retrospective study and permitted the publication of anonymized data.

## Additional Information

**How to cite this article**: Hann, A. *et al*. Feasibility of alternating induction and maintenance chemotherapy in pancreatic cancer. *Sci. Rep.* **7**, 41549; doi: 10.1038/srep41549 (2017).

**Publisher's note:** Springer Nature remains neutral with regard to jurisdictional claims in published maps and institutional affiliations.

## Supplementary Material

Supplementary Figure 1

## Figures and Tables

**Table 1:** Demographic baseline characteristics.

Characteristics	5FU/LV maintenance group	Control group
Patients	13	43
Age, mean	62	64
Age, range	48–73	47–79
Male	9 (69%)	27 (63%)
Female	4 (31%)	16 (37%)
ECOG 0–1	12 (100%)	43 (100%)
Tumor localization		
PDAC, head	7 (54%)	27 (63%)
PDAC, other	6 (46%)	16 (37%)
Disease stage		
Metachronous metastatic after initial surgery in curative intend with adjuvant gemcitabine	3 (23%)	11 (26%)
Synchronous metastatic	8 (62%)	28 (65%)
Locally advanced	2 (15%)	4 (9%)

**Table 2:** Number of chemotherapy cycles received by each patient in the 5FU/LV maintenance group during treatment course and duration of treatment pause.

Patient	Induction FOLFIRINOX	Induction FOLFOX	Maintenance 5FU/LV	Treatment pause (weeks)	Re-induction FOLFIRINOX	Re-induction FOLFOX
1	1	9	5	—	—	2
2	2	3	7	—	—	6
3	3	2	3	—	—	4
4	3	5	7	—	—	—
5	4	2	2	—	—	—
6	5	5	13	—	1	—
7	5	—	4	8.0	7	—
8	5	—	20	25.9	5	—
9	6	—	8	16.9	4	—
10	6	—	6	—	—	4
11	8	—	12	—	5	—
12	12	—	4	—	6	—
13	13	—	6	42.1	—	3

**Table 3:** Induction therapy.

Characteristic	
Patients	13
Treatment cycles, total	99
Treatment cycles, median (range)	6 (5–13)
Cycles containing	
FOLFIRINOX, median (range)	5 (1–13)
FOLFOX, median (range)	4 (2–9)
Cycles with	
Dose reduction	21 (21%)
Treatment delay ≥1 week	23 (23%)
Treatment time, median (range)	19.9 weeks (12.3–27.3)

**Table 4:** Maintenance therapy containing 5FU/LV.

Characteristic	
Patients	13
Treatment cycles, total	97
Treatment cycles, median (range)	6 (2–21)
Cycles with	
Dose reduction	9 (9%)
Treatment delay ≥1 week	16 (16%)
Treatment time, median (range)	14.1 weeks (4–54.1)

**Table 5:** Treatment pause.

Characteristic	
Patients	4
Pause duration in weeks, median (range)	21.4 (8–42.1)

**Table 6:** Re-induction therapy.

Characteristic	
Patients	11
Treatment cycles, total	47
Treatment cycles, median (range)	4 (1–7)
Cycles containing	
FOLFIRINOX, median (range)	5 (1–7)
FOLFOX, median (range)	4 (2–6)
Cycles with:	
Dose reduction	9 (19%)
Treatment delay ≥1 week	16 (34%)
Treatment time, median (range)	11.1 weeks (2–30.8)

**Table 7:** Adverse events.

Characteristic	
Adverse events leading to discontinuation, treatment delay or dose reduction/total cycles	48/243
Leukopenia	32 (67%)
Grade II	29
Grade III	3
Neuropathy grade II	7 (15%)
Nausea, emesis grade II	3 (6.3%)
Pleural effusion grade III	2 (4.2%)
Diarrhea grade II	2 (4.2%)
Biliary tract infection grade III	1 (2.1%)
Thrombocytopenia grade I	1 (2.1%)
Hospitalisations	3

## References

[b1] Conroy T. . FOLFIRINOX versus gemcitabine for metastatic pancreatic cancer. N. Engl. J. Med. 364, 1817–1825 (2011). 21561347 10.1056/NEJMoa1011923

[b2] Hann A., Bohle W., Egger J. & Zoller W. G. Improvement in advanced pancreatic cancer survival with novel chemotherapeutic strategies - experience of a community based hospital. Z. Gastroenterol. 54, 1138–1142 (2016). 27723905 10.1055/s-0042-110793

[b3] Tournigand C. . OPTIMOX1: a randomized study of FOLFOX4 or FOLFOX7 with oxaliplatin in a stop-and-Go fashion in advanced colorectal cancer–a GERCOR study. J. Clin. Oncol. 24, 394–400 (2006). 16421419 10.1200/JCO.2005.03.0106

[b4] Simkens L. H. J. . Maintenance treatment with capecitabine and bevacizumab in metastatic colorectal cancer (CAIRO3): a phase 3 randomised controlled trial of the Dutch Colorectal Cancer Group. Lancet Lond. Engl. 385, 1843–1852 (2015). 10.1016/S0140-6736(14)62004-3 25862517

[b5] Muñoz A. . Phase II study of bevacizumab, capecitabine, and oxaliplatin followed by bevacizumab plus erlotinib as first-line therapy in metastatic colorectal cancer. Oncol. Res. 21, 181–191 (2013). 24762224 10.3727/096504014X13887748696743

[b6] Hegewisch-Becker S. . Maintenance strategies after first-line oxaliplatin plus fluoropyrimidine plus bevacizumab for patients with metastatic colorectal cancer (AIO 0207): a randomised, non-inferiority, open-label, phase 3 trial. Lancet Oncol. 16, 1355–1369 (2015). 26361971 10.1016/S1470-2045(15)00042-X

[b7] Pox C. P. & Schmiegel W. [German S3-guideline colorectal carcinoma]. Dtsch. Med. Wochenschr. 1946 138, 2545 (2013). 10.1055/s-0033-1353953 24281967

[b8] Von Hoff D. D. . Increased survival in pancreatic cancer with nab-paclitaxel plus gemcitabine. N. Engl. J. Med. 369, 1691–1703 (2013). 24131140 10.1056/NEJMoa1304369 PMC4631139

[b9] Hosein P. J. . A retrospective study of neoadjuvant FOLFIRINOX in unresectable or borderline-resectable locally advanced pancreatic adenocarcinoma. BMC. Cancer 12, 199 (2012). 22642850 10.1186/1471-2407-12-199 PMC3404979

[b10] Kraemer P. C., Schmidt H. H. & Ladekarl M. Danish experiences with FOLFIRINOX as first-line therapy in patients with inoperable pancreatic cancer. Dan. Med. J 61, A4819 (2014). 24814594

[b11] Okusaka T. . Phase II study of FOLFIRINOX for chemotherapy-naive Japanese patients with metastatic pancreatic cancer. Cancer Sci 105, 1321–1326 (2014). 25117729 10.1111/cas.12501 PMC4462360

[b12] Mahaseth H. . Modified FOLFIRINOX regimen with improved safety and maintained efficacy in pancreatic adenocarcinoma. Pancreas 42, 1311–1315 (2013). 24152956 10.1097/MPA.0b013e31829e2006

[b13] Reure J. . Effectiveness and Tolerability of Maintenance Capecitabine Administrated to Patients with Metastatic Pancreatic Cancer Treated with First-Line FOLFIRINOX. Oncology 90, 261–266 (2016). 27097162 10.1159/000444854

[b14] Gebbia V. . Second-line chemotherapy in advanced pancreatic carcinoma: a multicenter survey of the Gruppo Oncologico Italia Meridionale on the activity and safety of the FOLFOX4 regimen in clinical practice. Ann. Oncol. Off. J. Eur. Soc. Med. Oncol. ESMO 18 Suppl 6, vi124–127 (2007). 10.1093/annonc/mdm240 17591805

[b15] Oettle H. . Second-line oxaliplatin, folinic acid, and fluorouracil versus folinic acid and fluorouracil alone for gemcitabine-refractory pancreatic cancer: outcomes from the CONKO-003 trial. J. Clin. Oncol. Off. J. Am. Soc. Clin. Oncol. 32, 2423–2429 (2014). 10.1200/JCO.2013.53.6995 24982456

[b16] Golan T. . Overall survival and clinical characteristics of pancreatic cancer in BRCA mutation carriers. Br. J. Cancer 111, 1132–1138 (2014). 25072261 10.1038/bjc.2014.418 PMC4453851

[b17] de Gramont A. . Randomized trial comparing monthly low-dose leucovorin and fluorouracil bolus with bimonthly high-dose leucovorin and fluorouracil bolus plus continuous infusion for advanced colorectal cancer: a French intergroup study. J. Clin. Oncol. Off. J. Am. Soc. Clin. Oncol. 15, 808–815 (1997). 10.1200/JCO.1997.15.2.808 9053508

