# Supplementary material for: Feasibility of alternating induction and maintenance chemotherapy in pancreatic cancer
Source: Sci Rep. 2017 Jan 31;7:41549. doi: 10.1038/srep41549 (PMC5282479; doi:10.1038/srep41549)
Supplement: Supplementary Figure 1 [file srep41549-s1.doc]

**Title**

Feasibility of alternating induction and maintenance chemotherapy in pancreatic cancer.

**Authors**

Alexander Hann^1,2*^, Wolfram Bohle^1^, Jan Egger^3^, Wolfram Zoller^1^

1 Department of General Internal Medicine and Gastroenterology, Katharinenhospital, Stuttgart, Germany

2 Department of Internal Medicine I, Ulm University, Ulm, Germany

3 Institute for Computer Graphics and Vision, Graz University of Technology, Austria

* corresponding author, [alexander.hann@uniklinik-ulm.de](mailto:alexander.hann@uniklinik-ulm.de)

**Corresponding author:**

Dr. med. Alexander Hann

Uniklinik Ulm

Klinik für Innere Medizin I

Albert-Einstein-Allee 23

89081 Ulm

Email: alexander.hann@uniklinik-ulm.de

Tel.: 0049 731 / 500 44750

Fax: 0049 731 / 500 44502

**Supplementary figure**


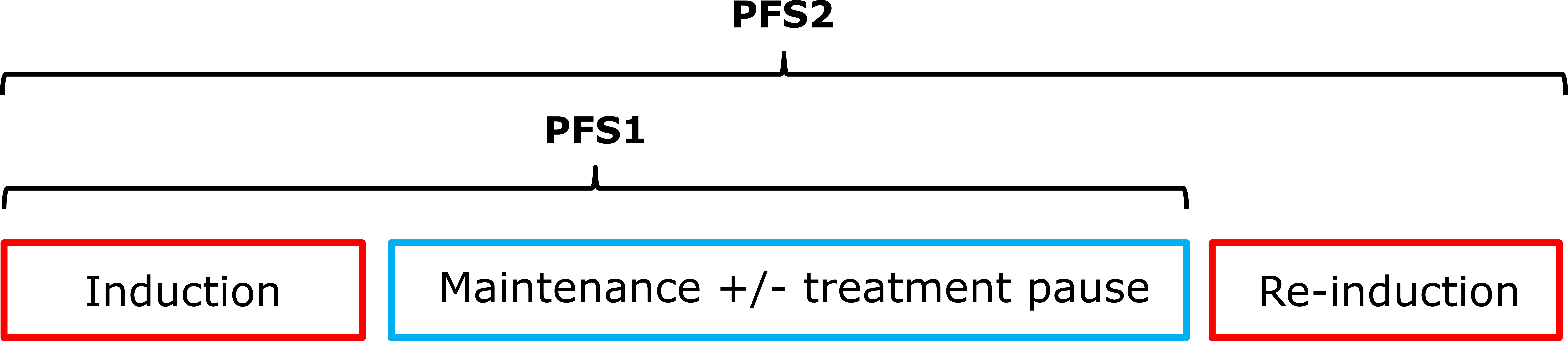


Supplementary figure 1: Definition of progression free survival (PFS) 1 and 2.
